# Toward IoT fog computing-enabled system energy consumption modeling and optimization by adaptive TCP/IP protocol

**DOI:** 10.7717/peerj-cs.653

**Published:** 2021-08-05

**Authors:** Aladdin Masri, Muhannad Al-Jabi

**Affiliations:** Computer Engineering Department, An-Najah National University, Nablus, Palestine

**Keywords:** IoT, Wireless networks, TCP, Energy consumption, MTU size, FOG computing

## Abstract

Nowadays, due to the fast-growing wireless technologies and delay-sensitive applications, Internet of things (IoT) and fog computing will assemble the paradigm Fog of IoT. Since the spread of fog computing, the optimum design of networking and computing resources over the wireless access network would play a vital role in the empower of computing-intensive and delay-sensitive applications under the extent of the energy-limited wireless Fog of IoT. Such applications consume considarable amount of energy when sending and receiving data. Although there many approaches to attain energy efficiency already exist, few of them address the TCP protocol or the MTU size. In this work, we present an effective model to reduce energy consumption. Initially, we measured the consumed energy based on the actual parameters and real traffic for different values of MTU. After that, the work is generalized to estimate the energy consumption for the whole network for different values of its parameters. The experiments were made on different devices and by using different techniques. The results show clearly an inverse proportional relationship between the MTU size and the amount of the consumed energy. The results are promising and can be merged with the existing work to get the optimal solution to reduce the energy consumption in IoT and wireless networks.

## Introduction

The Internet of things (IoT) is a trend in the field of wireless communication in which many smart devices are involved in sharing information, collaborative decision making, and optimizing the task accomplishment time. Gartner projects more than 50 billion devices will be connected to the network by the year 2025. Moreover, IoT is all about data collection, data usage, communication among devices and with the world. Therefore, big data require extensive storage capacity, cloud computing, and interminable channel bandwidth for transmission which makes IoT omnipresent. Nevertheless, big data communication consumes a significant amount of power (Dange & Chatterjee, 2020) such that the long transmission from IoT devices to the cloud servers may cause delay fluctuation and appeal to extra transmission energy cost.

Consequently, to serve the IoT applications, the computing resources need to be chosen upon the heterogeneity of the IoT devices. Because of the different constraints of IoT devices, resource provisioning in the cloud is not a trivial task. Therefore, fog computing is the appropriate platform to deal with such constraints of IoT, such that fog computing can address different IoT challenges like scalability, heterogeneity, and low latency by adapting intelligent features of machine learning in its resource management techniques (Gowri & Bala, 2019).

In addition, since the IoT big data streams are transmitted to the cloud in high volume and at a fast velocity, it is necessary to design an efficient data processing architecture to explore the valuable information in real-time (Sun & Ansari, 2016; Ai, Peng & Zhang, 2018). Therefore, due to the incoming 5G era, there is an increasing demand for the integration with fog computing (FG), resource virtualization (RV), and broadband wireless internet to speed up the transfer of the IoT perception from the theoretical position to the practical implementation (Goudarzi et al., 2021; Subramanya, Harutyunyan & Riggio, 2020).

**Figure 1:**
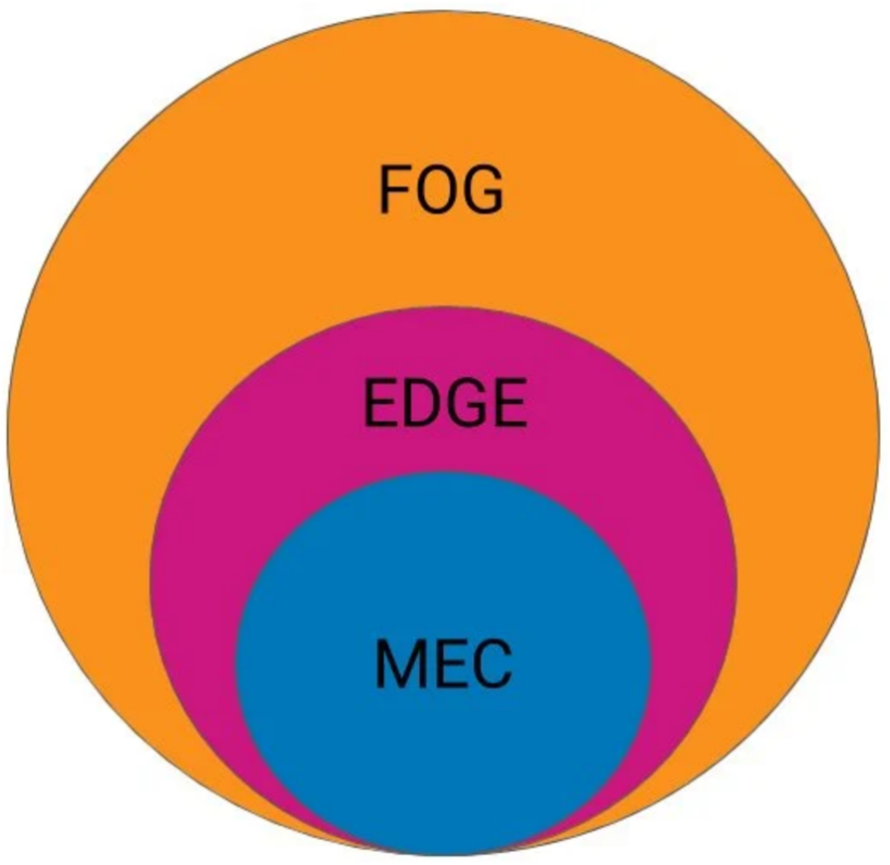
Comparison between Fog, EDGE and MEC.

Fog computing is defined as the architecture that distributes computing, control, networking, and storage resources and services anywhere far from the cloud and closer to things. As shown in Fig. 1, fog computing is the global architecture of distributing resources across the network, whereas edge computing is an underlying term that focuses on executing compute processes close to end-users outside the network core, and mobile edge computing (MEC) is an architecture standard for edge computing. Thereafter, MEC architectures successfully solve the problem of high latency in cloud computing because the infrastructure-based cloud servers are always located centrally in the core network and far away from the IoT devices (Wei, Luo & Sun, 2020).

Although MEC has fewer resources as compared to the cloud, MEC not only provides the computational facility but also the storage and caching services at the edge of mobile devices (Mahmood et al., 2020). MEC is conceptualized as an auspicious approach to improve offloading efficiency. In the MEC framework, cloud computing means are equipped within the radio access network nearby these mobile devices. In other words, with the cooperation of MEC, IoT devices are empowered to migrate their tasks to the MEC servers on the edge of the network, rather than exploiting the servers in the core network (Subramanya, Harutyunyan & Riggio, 2020). Consequently, the Cloudlet paradigm is introduced such that the cloudlets are based on dedicated devices with capacities similar to a data center but at lower scale present close to the consumers. This paradigm allows end devices to offload computing to the Cloudlet devices with resource provisioning similar to that of a data center (Ai, Peng & Zhang, 2018; Idrees & Al-Qurabat, 2020).

**Figure 2:**
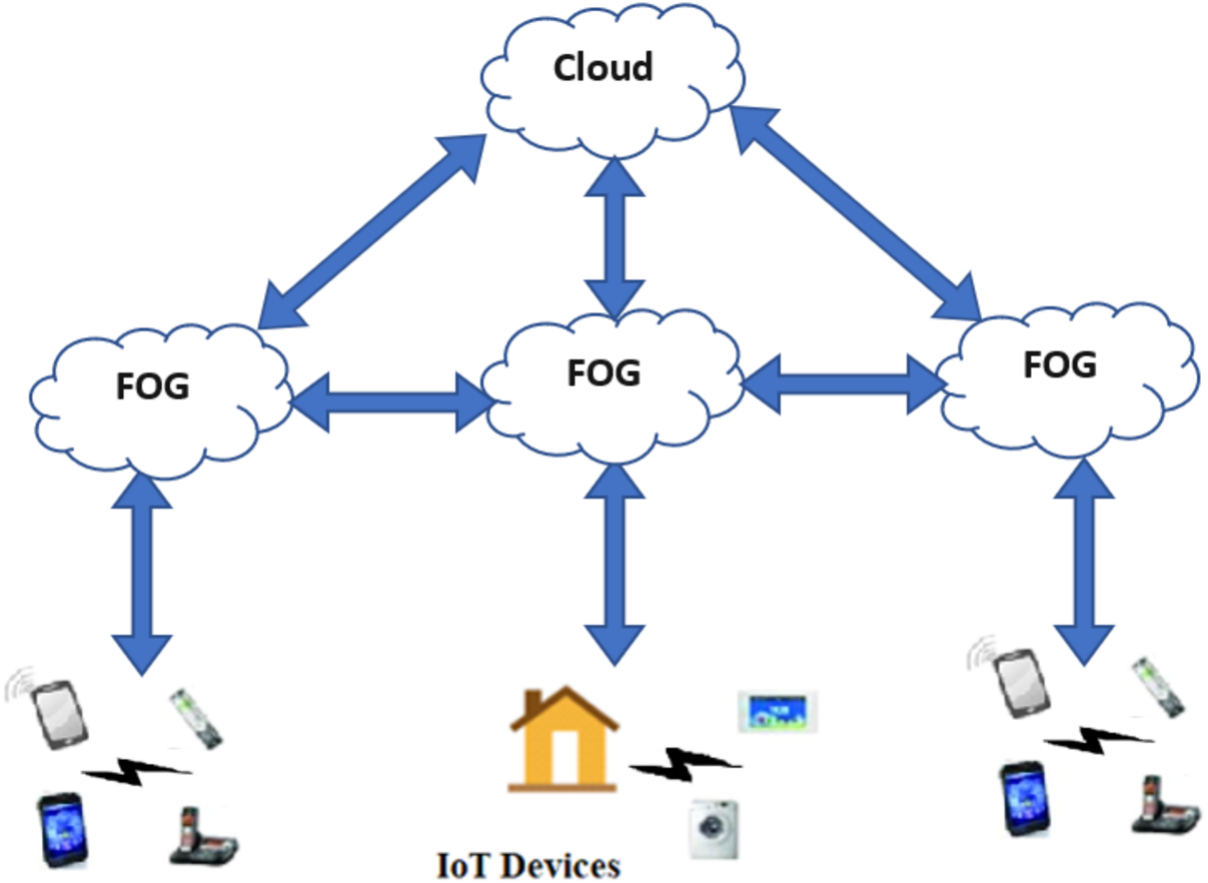
IoT Fog computing-enabled system.

The architecture of an IoT Fog computing-enabled system, as shown in Fig. 2, consists of three levels: the data collection level which contains the end devices that collect data, the processing level that represents the cloud, and the intermediate level that contains the interconnected fog elements and IoT gateways that respond to end devices in real-time and exchange messages with end devices and with the cloud.

It is usually evident that fog elements and IoT gateways need to be connected to the electrical grid; however, in many cases such as in time-sensitive applications like natural disaster rescue operations or natural evidence monitoring, IoT nodes and gateways are commonly deployed in large areas, such that natural energy sources like solar or wind can be used to charge the batteries that power every device (Suárez-Albela et al., 2017; Yazdinejad et al., 2020). Also, these elements consume a significant amount of energy to execute different tasks. Eventually, this will pose a challenge soon to minimize energy consumption and will also demand new ways of developing low energy communication across the network (Zeadally et al., 2020). One of the assertive obstacles in implementing IoT fog computing-enabled schemes is supplying sufficient energy to operate the network without endangering the quality of service (Kamalinejad et al., 2015). It is imperative to improve the energy efficiency and longevity of elements in such systems. Motivated by the fact that the Transmission Control Protocol (TCP)/Internet Protocol (IP) suite is the existing standard nowadays for computer communications in the networked world, IP-based solutions could be the future for networks that construct IoT fog computing-enabled system (Sheng et al., 2013).

Because of the tremendous amount of data transfered in IoT fog computing-enabled systems, the communication in these systems consumes a significant amount of energy. Therefore, efficient protocol selection and optimization play a vital role in energy conservation in such systems. The TCP protocol is an efficient one that consists of different features that organize its functionalities and characters. One of the most interesting features of the TCP protocol is the size of the Maximum Transmission Unit (MTU). The MTU has a big role in fragmenting the data into packets, and as big the MTU as the maximum packet size can be achieved. In wireless networks, the packet count is also important because of the calculations and operations that the machine must do to send these packets. However, the energy source for the devices that form the network is limited; the higher energy consumed, the smaller the life of that device.

In this article, the work focuses on minimizing the energy consumed by the IoT Fog computing-enabled systems. Given that the computation system is distributed in many locations (devices, edge, cloud, etc.), there is a high volume of data exchange. Therefore, it is highly critical to keep the node devices operating. The novelty of our work is the optimization of the MTU size in TCP protocol to get optimum results. The MTU size is modified in the header as a TCP parameter. The following sections of this paper include: related work in “Literature Review”, the methodology and the proposed architecture are discussed in section “Methodology”. The “Measuring Procedure” section presents the complete steps used to perform the experiments, and in the section “Experiments and Results” the detailed experiments are described and the results are discussed. Finally, the “Conclusion” section contains the conclusion of this work.

## Literature Review

Wireless device energy plays a vital role in the sustainability of device functionality. Therefore, efficient energy consumption in wireless devices attracted many researchers such that (Mahmoud & A. Mohamad, 2016), presented a study of IoT application power consumption in different wireless technologies. The study focused on introducing a comparison between different low-power wireless communication technologies such as low-power Wi-Fi, 6LowPAN, LPWA, ZigBee, and their modules in power conservation as well as in the extension for the life of the IoT network sensors. The results of the study showed that different module for each protocol consumes a different amount of energy. Therefore, protocol module selection plays a vital role in saving battery life. In other words, the assessment of protocols with each other relies on the module used. Pötsch, Berger & Springer (2017) presented a measurement solution for tracking the highly dynamic power consumption of wireless embedded systems. Different use cases were presented to demonstrate the usability and simplicity of the power measurement system.

In Sun et al. (2014), the authors presented a measurement study of smartphones’ WiFi active energy modeling focusing on the application layer through the models where parameters are easily available to developers. In Carroll & Heiser (2010), a power model of the Freerunner device was developed and the energy usage and battery lifetime under some usage patterns were analyzed. Also, the energy impact of dynamic voltage and frequency scaling of the device’s application processor was analyzed. (Shen, 2016) presented an adaptive power controllable WiFi adjusting method and device.

In Wang, Hempstead & Yang (2006), a realistic power consumption model for WSN devices was developed by consolidating the characteristics of a typical low-power transceiver. And a comparison between single-hop and multi-hop routing schemes was performed based on the power consumption model.

On the network layer, Dunkels et al. (2012) presented the networking capabilities of IPv6 as well as its support in an operating system called Contiki which is the first operating system that introduced the low-power wireless systems IP networking perception. Also, the authors discussed the implementation, the design, and the low-power evaluation mechanisms of IPv6 in Contiki.

On the other hand, different researchers worked on the transport layer. Qiu & Lee (2011) presented a method and system for transmission control packet segmentation offload. If the packet is identified as a large send offload packet (LSOP), a selection may be made between handling the packet through a hardware process or a firmware process. The packet is processed by either the selected hardware process or the firmware process. In Shang, Yu & Droms (2016), the technical challenges in applying TCP/IP to the IoT environment were analyzed and various solutions were proposed by the IETF were reviewed. The authors argued that existing IP-based solutions are either incompetent or inadequate in supporting IoT applications and that a more effective solution would grasp the information significant network architecture. However, in Gomez, Arcia-Moret & Crowcroft (2018), the authors argued that the current directions predict that TCP will have a vast advancement in IoT network schemes despite it was commonly ignored in the designs of IoT networks.

On the other hand, some researchers discussed high-level issues in fog computing such as in Tange et al. (2020), Baniata, Anaqreh & Kertesz (2021), He et al. (2020), Luong et al. (2020), Xiong et al. (2019) and Martinez, Hafid & Jarray (2020) focused on privacy, security, and the optimization task allocation and resource management issues in fog computing, while Essalhi, El Fenni & Chafnaji (2020) proposed an approach to ensure smart energy management during processing and communication of offloaded tasks in fog applications taking in consideration the latency constraints and Raj & Basar (2019) proposed a methodology to determine the shortest route to provide energy-efficient and enhanced routing capabilities for IoT with WSN; also, Chen, Ma & Liu (2018) presented an algorithm for IoT in e-Healthcare to decide the optimal packet size by either minimizing the delay at a prescribed transmission power or minimizing energy consumption with a prescribed delay.

Therefore, it is clear that there is a shortage in existing literature where none of the researches dealt with energy conservation through optimizing one of the parameters for the existing protocols. However, this work is focusing on optimizing energy consumption in IoT fog computing-enabled systems through optimizing the TCP’s MTU size.

## Methodology

As aforementioned, the main contribution of our work is to develop a model to estimate the consumed energy during communication operations in wireless devices to decrease it in such devices. Since the wireless devices have limited battery size, their lifetime is directly affected by the consumed energy. To increase the battery life, different techniques can be used. This includes the use of bigger batteries, making the device sleep, idle, or making modifications on the MAC layer to increase its efficiency. Furthermore, the TCP/IP protocol parameters can simply be adjusted.

The TCP’s MTU size can be modified to different values for the same machine. The maximum MTU size for wired machines is 1,500 bytes, while for wireless networks, it can be augmented to reach 2,312 bytes (802.11). Changing the MTU value affects the maximum size of the sent packet (the sum of sent data plus the headers), and so, the number of sent packets. When the machine sends a packet, it makes different calculations and operations that cost a lot of energy for the wireless device which has a limited energy source. Therefore, it is important to observe how the energy consumption amount will be affected when the machine sends smaller or larger packets.

The goal of this work is how to conserve the devices’ energy for a longer time. So, the following steps show the used procedure for measuring the consumed energy:

(1) Measuring the consumed energy under different conditions:

Let us first mention the components of the total energy consumed by the machine:

The first component is the energy consumed by the machine itself or by the system. such that, this energy is the electrical energy spent by the system with or without transmission. Hence, this energy is reserved.

The second component is the energy used in the sending or receiving operations. Fragmentation, sequence number, sum check, destination or target one or more, etc. are done when the machine wants to send data. When it receives a packet, it checks the received packets and the sequence number, sends an acknowledgment, etc.

Different methods can be used to measure the consumed energy. One method is to use mathematical equations, linear or complex ones. However, these equations are sometimes difficult to implement, or they may ignore some important points in real communication. In addition, these equations do not allow the calculation of the energy consumed by the machine itself and the energy consumed during communication separately. Furthermore, the following points show some mathematical equations that can be used to measure the consumed energy:

• Using simple linear equations

\begin{eqnarray*}Energy=m\ast s+b \end{eqnarray*}

where *m* and *b* are variant coefficients, *s* is packet size.

However, this equation does not consider several impacts, like fragmentation, bit error, connectivity, etc.

• Using complex equations

\begin{eqnarray*}{E}_{packet}=[{P}_{transmit}+{P}_{electonic}].{T}_{bit}.[L+H]+{E}_{overhead} \end{eqnarray*}

Thus, as the linear equation, it does not consider some issues like interference, acknowledgment process, etc.

Along with that, the other method to measure the total energy consumed was by putting the machine under different conditions and using different applications and platforms, such as:

- Changing the MTU size to observe its effect on energy consumption
- Using different applications; ftp (file transfer protocol), Ping protocol, and a java program to send and receive a continuous stream of dummy data.
- Using different platforms and devices: core i7 laptop, mobiles of different processors and brands, PDAs, temperature sensors, humidity sensors, etc.

(2) Measuring the energy consumption under real traffic and network:

To estimate the consumed energy under normal conditions and in a real network, the devices were put to operate in normal use. With the help of the Linux platform, the real packets transmitted or received could be captured. Once more, the measurements were held for different sizes of MTU. Thus, the energy consumed in communication with real traffic can be calculated for a given MTU size.

(3) Deriving a transformation algorithm to estimate the effect of changing the MTU size in the whole network:

The last step in the experiments was to observe the effect of changing the MTU size for all devices to a given value. Nevertheless, this part can be only done for an MTU of 1,500 bytes, since the MTU size for all the network devices cannot be changed. So, from the results of the previous steps, a mathematical algorithm can be derived which can be used to estimate how the total traffic in a network would be for different values of MTU.

**Figure 3:**
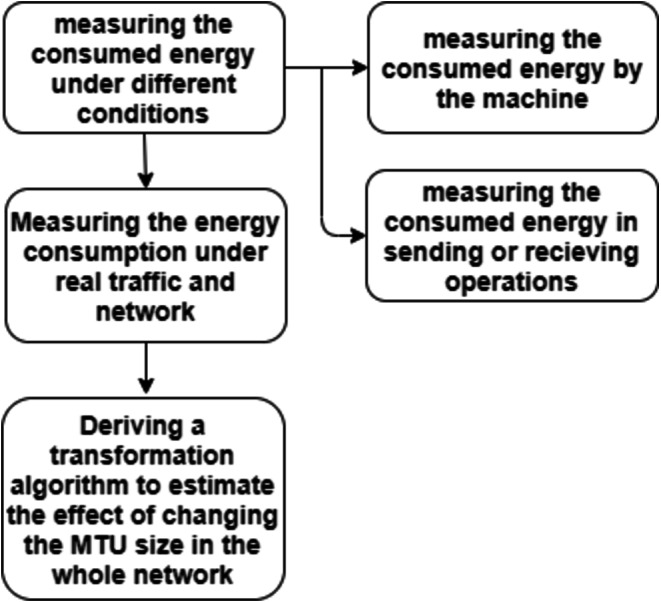
Procedure for measuring the consumed energy.

Figure 3 represents a flowchart of the procedure used for measuring the consumed energy.

**Figure 4:**
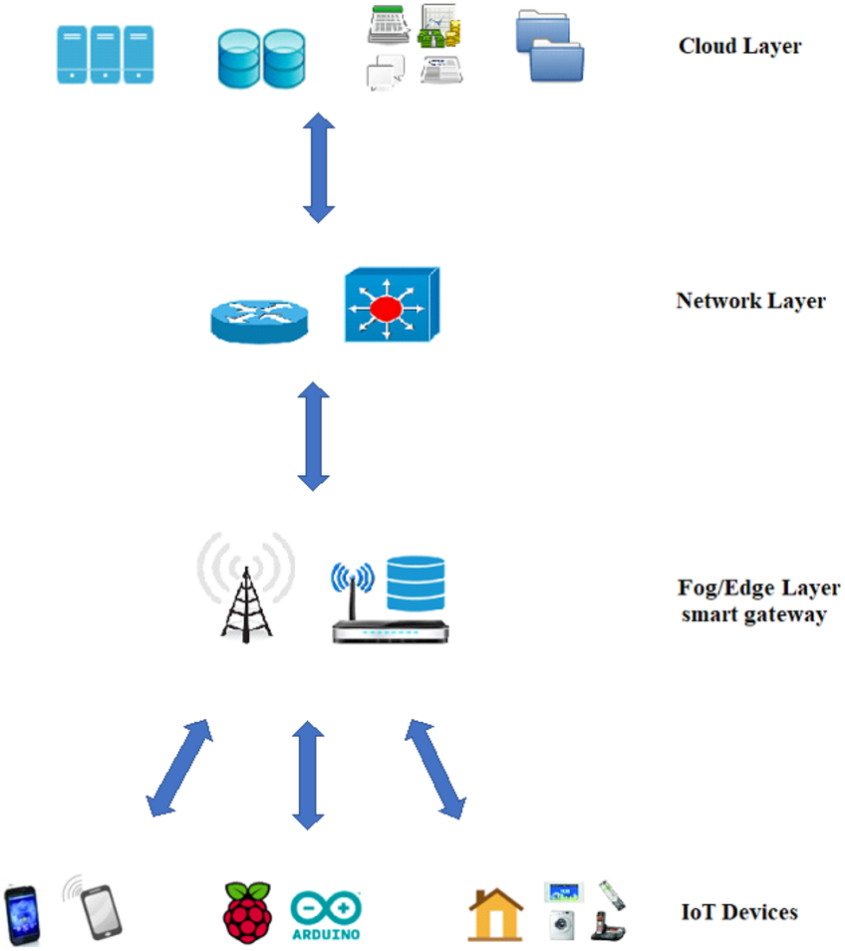
Communication between the different layers and IoT devices.

One of the widely spread environments, in which the communication energy-saving via TCP-MTU size plays a vital role in saving the total consumed energy, is the fog computing environment. Figure 4 clearly shows the communication between the different layers and how IoT devices exchange information across the fog edges and the network layer to reach the cloud layer. Here, the amount of energy saving is important. Also, the use of TCP IP protocol is vital.

## Measuring Procedure

Measuring the consumed energy in a machine when it sends or receives data depends on two factors: the amount of consumed energy or the needed time to send or receive a given data and the amount of that data. By considering these two factors, the relationship between the energy consumption by the machine and the variation in the conditions can be figured out through simple mathematical manipulation.

### Used applications to realize the experiments

1- FTP, file transfer protocol, RFC 959. FTP was used to transmit a continuous stream of data, to see its effect on the energy consumed in sending data.
2- A Java program was used as an alternative to the FTP protocol. The same idea of sending a continuous stream of data to see its effect on the energy consumed in sending data. As the FTP protocol, client–server programs were used on both machines. Another use of Java was in measuring the silence time, where a do-nothing program was used.
3- Ping, Packet Internet Groper. A ping storm is a condition in which the internet ping program is used to send a flood of packets to a machine to test its ability to handle a high amount of traffic. This is known in ping options as –*f* option, which was used in this work. Some other options, like –*s* to define the size of packets or –*c* to define the count of echo request packets, were also used.

Note: the used system is Linux Ubuntu, and to use the –*c* option user must be root to define the count to be more than three packets.

### Measuring principles

The following steps explain the used techniques to measure the consumed energy:

1- Measuring the silence time that is the time in which the machine does not send or receive any data. To do the experimentation, either the time or the consumed energy must be fixed to a certain value. In this work, the energy consumed was always 25% of the full battery energy. The battery was charged until 100% and the measured values were until the battery reached 75% (25% of energy was consumed).
2- Measuring the consumed energy from the system itself. This was done by deactivating the transmission antenna to ensure that no data is sent or received. To accomplish this point, a do-nothing java program was turned on the machine.
3- The program was left running until the device dropped from 75% to 25% of the battery level. These values were chosen to satisfy the recommendations of the manufacturer. Hence, the minimum recommended charge level is 20% to prevent fast drainage (Hannan et al., 2018). Also, all the employed devices have new batteries (less than six months).
4- To measure the energy consumed in sending and receiving a byte, Ping and ftp protocols were used for a continuous stream of data. In addition, the time spent to reach the required battery level was measured. Here, to calculate the value of the consumed energy for sending or receiving a byte, the energy consumed from the system must be subtracted from the total energy measured for each case. Note that consumed energy is a function of streamed data and time.
5- The measurements were repeated many times, and for different MTU sizes, to get an average value for each device.

## Experiments and Results

Over most of the communication networks, the normal MTU size is 1,500 bytes. Changing the MTU size will surely affect the packet size of the data that will be sent. Therefore, when an MTU of 1,500 bytes is used, the maximum packet size will be 1,514 bytes of data and headers together. However, if the MTU size is decreased to 1,000 bytes, the maximum packet size will be 1,014 bytes of data and headers together.

Thus, if the data stream is 10,000 bytes, and the MTU size is 1,500 bytes, then there will be six packets of 1,514 bytes and one packet of 1,210 bytes, with a header average size of 42 bytes. However, if the same amount of data is sent using an MTU of 1,000 bytes, there will be 10 packets of 1,014 bytes and one packet of 322 bytes. This means the machine will send more packets for the same amount of data, 11 packets instead of seven. Therefore, the machine will make more calculations and consume much more energy for the same amount of data. Not to forget, more time is needed to send 11 packets instead of seven packets. In addition, not to forget that a packet of 1,500 bytes is greater than one of 1,000 bytes.

In our work, three different values of MTU were used each time for each experiment: 1,500, 1,000, and 500 bytes. Note: any other MTU size can be used.

### A. Measuring the consumed energy

(1) Silence Time Consumed Energy: The silence time is the time where the machine does not send or receive any data. The idea here is to measure the time needed for the machine to reach 75% or to consume 25% of the full battery energy. The silence time does not depend on the size of the MTU because no sent or received data or antenna is working. So, for all values of MTU, the silence time will always be the same. Firstly, the battery was fully charged. Then, a do-nothing Java program was turned on the machine. When the battery value reached 75% of the full energy, the time was measured.

**Definition**: The system energy consumption S, can be expressed in the battery unit per second, as in (1).

(1) \begin{eqnarray*}S= \frac{25}{{d}_{s}} \end{eqnarray*}

where *d*_*s*_ is the time that the system needs to reach 75% of the full battery for a given MTU size. Table 1 shows the measured time.

**Table 1:** The value of ds for different values of MTU.

MTU size (byte)	**d**_**s**_ **(m) (sec)**
1500	3420
1000	3530
500	3385
Avg.	3445

The average value of S is:

\begin{eqnarray*}S= \frac{25}{{d}_{s}} = \frac{25}{3445} =7.26E-3~\mathrm{ub}/\mathrm{s} \end{eqnarray*}

(2) Transmission Consumed Energy:

The energy consumption in transmitting data is the energy consumed in sending continuous data until reaching 75% of the fully charged battery energy. To measure this value, FTP protocol and a java program were used. A client/server method is used, where one machine was set as a server and another one as a client.

The first step was by opening a connection channel, then a continuous stream of data was sent from the wireless machine to the other machine. The wireless machine was left until the battery reached 75% of the full battery energy. Both the elapsed time and the sent data were measured in each trial. The experiment was held for different values of MTU; 1,500, 1,000, and 500 bytes size. As a result, the energy consumption by the transmission *t* (*m*) function for FTP or Java is calculated. Hence: the energy consumed by the system itself must be subtracted from the resulted values.

**Figure 5:**
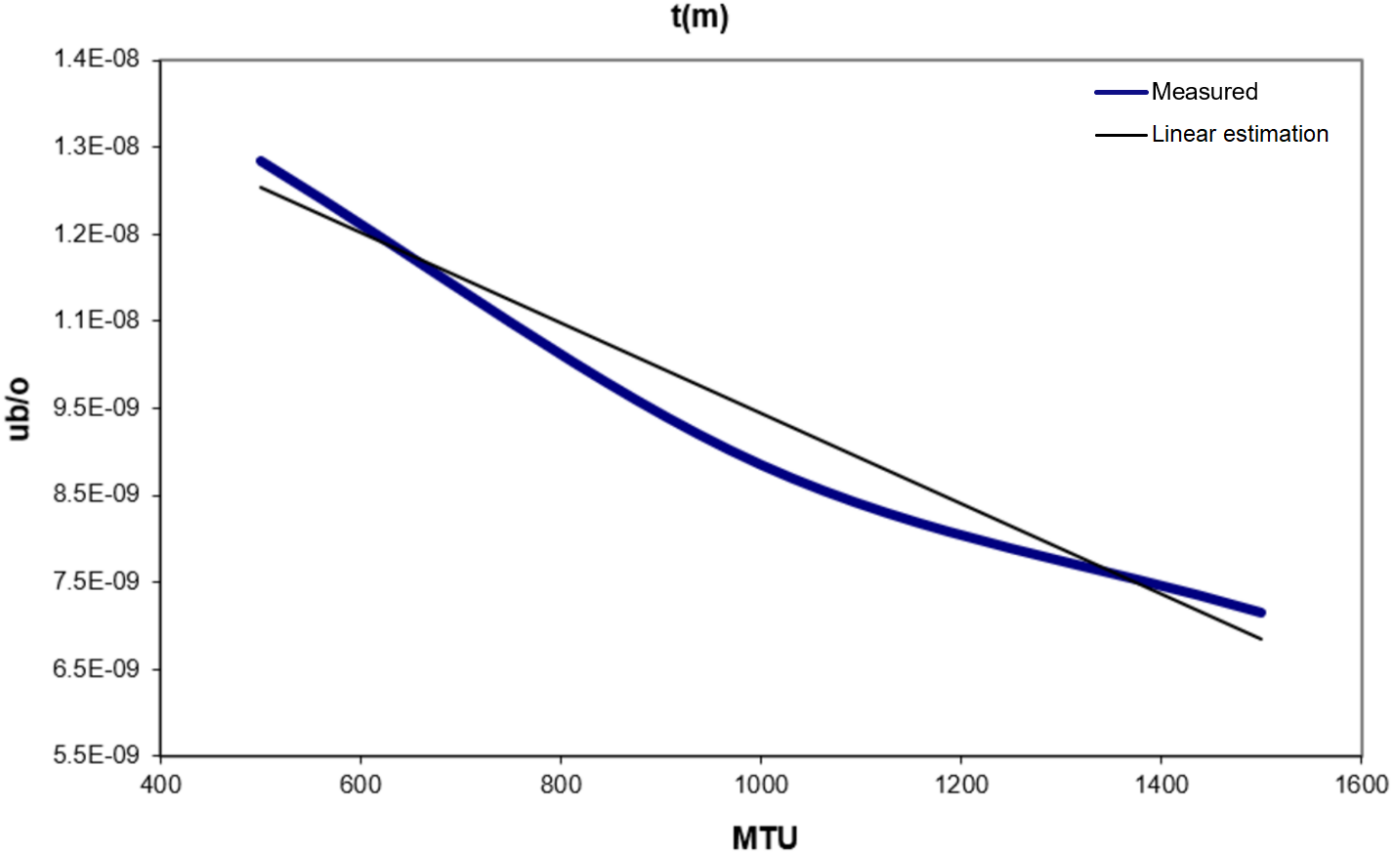
T(m) vs. MTU.

Table 2 shows the values of *d*_*f*_(*m*), *dj* (*m*), *q*_*f*_(*m*), and *q*_*j*_(*m*) for different sizes of MTU. Where *d*_*f*_(*m*) and *d*_*j*_(*m*) are time in seconds, while *q*_*f*_(*m*) and *q*_*j*_(*m*) are the quantity of sent data in bytes. Table 3 and Fig. 5 show the variation of energy consumed for different values of MTU.

**Table 2:** The values of d_f_(m), d_j_(m), q_f_(m) and q_j_(m).

**MTU**	**1500**	**1000**	**500**
**FTP**			
**d**_**f**_ **(m) (sec)**	2106.54	2217.13	2285.75
**q**_**f**_ **(m) (byte)**	1357905920	1006632960	681574400
**Java**			
**MTU**	**1500**	**1000**	**500**
**d**_**j**_ **(m) (sec)**	2110.51	2218.85	2285.71
**q**_**j**_ **(m) (byte)**	1315962880	1006632960	681574400

**Table 3:** t(m) for different values of MTU.

**MTU**	**t(m) (ub/octet)**
1500	7.15297E−09
1000	8.85181E−09
500	1.23428E−08

**Definition**: The consumption in transmission function *t* (*m*) is the energy needed to transmit one byte. It is expressed as a battery unit per byte (octet). From the measured values, the function of *t* (*m*) can be expressed as follows:

(2) \begin{eqnarray*}t \left( m \right) = \frac{25-S.{d}_{x} \left( m \right) }{{q}_{x} \left( m \right) } \end{eqnarray*}

From Figure 5, a linear equation for the consumed energy in transmitting one byte can be estimated:

(3) \begin{eqnarray*}t(m)=-5.190E-12\ast m+1.464E-08\end{eqnarray*}

(3) Reception Consumed Energy:

The reception consumed energy is the energy consumed in receiving a continuous stream of data until the battery reaches 75% of its full charge. To realize this experiment, the Ping program was used. In the Ping program, the echo request size equals that of the echo-response. In other words, if a packet of 1,000 bytes was sent as an echo request from one machine, the other machine will reply by sending another packet of 1,000 bytes as an echo response.

Repeatedly, the experiment was to measure the elapsed time and the sent data when the consumed energy is 25%. Table 4 shows the results of this experiment.

**Table 4:** The use of Ping to measure the time and sent data.

**Ping**			
**MTU**	**1500**	**1000**	**500**
**Time d**_**j**_ **(m) (sec)**	2245.71	2290.25	2335.16
**Quantity q**_**j**_ **(m) (byte)**	501952000	432540000	330966400

Since the consumed energy in sending one byte and the silence time are already known, and it is known that the sent data equals the received data, then this information can be applied with the measured values to obtain the consumption in reception *r* (*m*) as follows in Eq. (4):

(4) \begin{eqnarray*}r \left( m \right) = \frac{25-S.{d}_{p} \left( m \right) -{q}_{p} \left( m \right) .t \left( m \right) }{{q}_{p} \left( m \right) } \end{eqnarray*}

**Definition:** The consumption in reception function *r* (*m*) is the energy needed to receive one byte.

**Figure 6:**
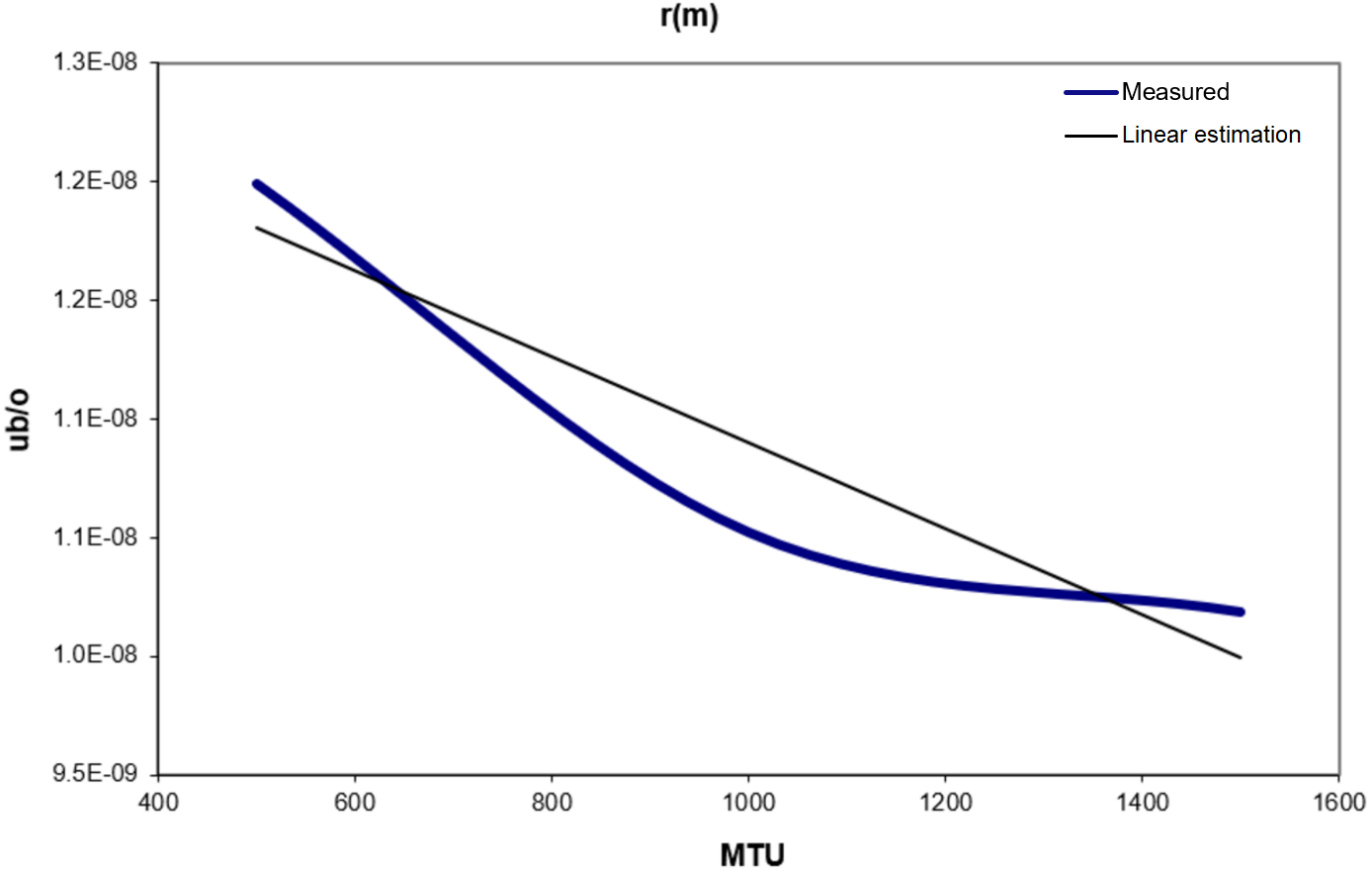
R(m) vs. MTU.

Table 5 and Fig. 6 show the values of *r* (*m*). From Fig. 6 a linear estimation equation for *r* (*m*) can be obtained:

(5) \begin{eqnarray*}{r(m)=-1.806\ast 10}^{-12}{\ast m+1.271\ast 10}^{-8}ub/o\end{eqnarray*}

**Table 5:** r(m) for different values of MTU.

**MTU**	**r(m) (ub/octet)**
1500	1.01856E−08
1000	1.05219E−08
500	1.19919E−08

### B. Energy consumption estimation and real traffic

In the previous part, the whole work was performed for a continuous data stream with a predefined MTU value for one device. However, in real traffic, the received and sent packets are not always continuous, and their size varies over time.

Nevertheless, the following points have to be considered in real traffic:

a- The Internet is usually not used for a long time.
b- Even when the Internet is used for a long time, the wireless device often receives or sends little data during this time.
c- Most of the received packets are in the form of acknowledgment or a broadcast, and most of them have a small size.

These points summarize the major differences between real traffic and continuous data stream.

In the next part, it is considered that the user would use the Internet not more than 25% of the total usage of his machine. The rest of the usage would be for other things; idle, exchanging control messages, etc. In the case of sensor networks, long periods could separate between occurrences of important data.

#### Global traffic.

The global traffic *g* (*p*) is the number of packets of a given size per second (or unit of time), where *p* is the packet size. The number of packets of a given size can be zero or more in a given period depending on the traffic itself and the MTU value. In addition, the machine would not send or receive any packet greater than the MTU in size. If the MTU size was 1,500 bytes (which is the default value), there is no packet of size greater than 1,500 bytes. However, if the value of MTU is changed to a new value *p*, the machine would not receive or send packets greater than *p* bytes.

In this part, the global traffic was observed by the use of the Linux command *tcpdump* with option *–e*. This command allows observing traffic for a given machine, and the –*e* option prints the link-level header on each dump line. Nevertheless, the experiment can be done only for an MTU size of 1,500 bytes. This is because the MTU size is by default configured to 1,500 bytes for the machines. Thus, it is difficult even impossible to change the size of MTU for all the machines in the network.

**Figure 7:**
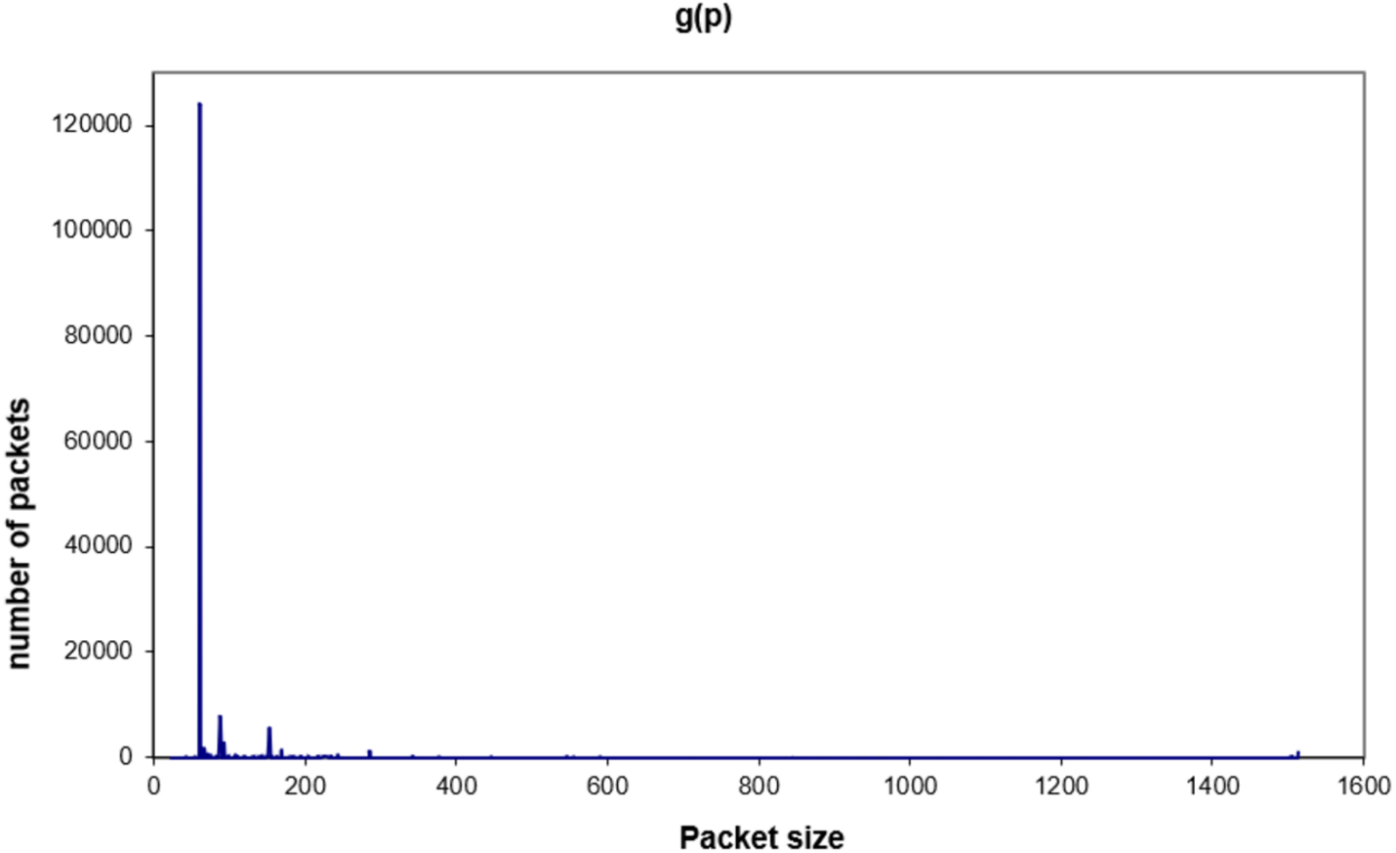
Global traffic for a 1,500-byte MTU.

To get the real packets in the network, the experiments were held for one hour each, and with the help of *tcpdump*, *grep* and *sed* commands. Figure 7 shows the global traffic (packets received and sent) over the network for one hour and an MTU size of 1,500 bytes. The distribution shows clearly that most of the packets are of small size and in the range 60–160 bytes (nearly 64% of the packets are 60 bytes size). This huge number of small packets would cost the device more energy. This is obvious as shown in Figs. 5 and 6 and with the linear Eqs. (3) and (5). In other words, the consumed energy is proportional to the number of small packets.

By taking a deep look at these packets, it is noticed that the majority of these packets were: Broadcast / arp whohas, acknowledgment, or LLC. These packets could not be neglected or eliminated because they are control packets. Moreover, they will be increased in case the size of MTU is decreased because there will be more received packets and there is a need to send more control packets (especially acknowledgment packets). The next part will focus on this issue.

#### Transmitted traffic by one machine.

**Figure 8:**
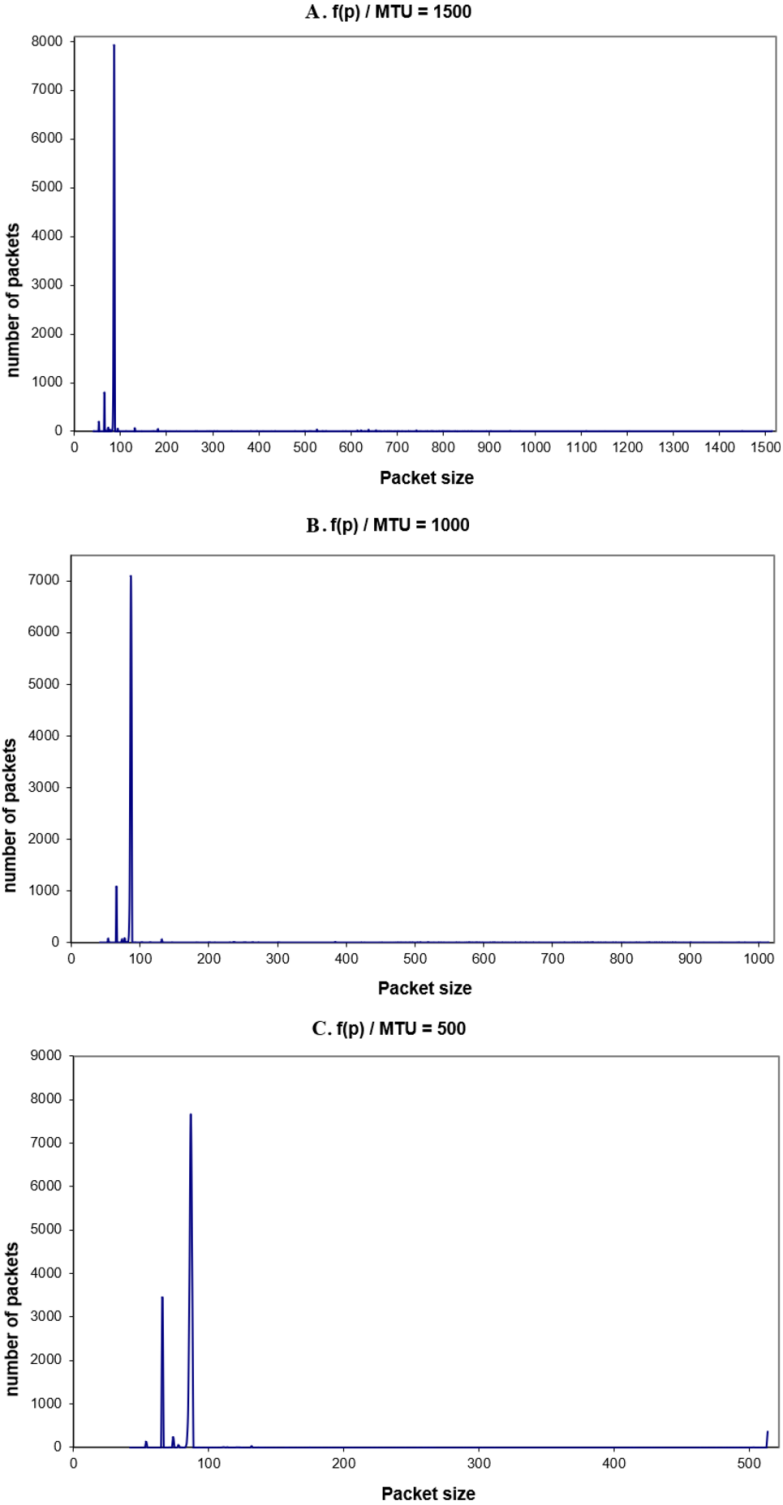
(A–C) F(p) for different values of MTU.

The transmitted traffic by one machine *f* (*p*) is the number of packets of a given size per second, (or unit of time), or the packets that are sent by one machine only. The main difference between the global traffic and this traffic is that the size of MTU can be changed for that machine for different values. This helps to observe the transmitted traffic for other MTUs than the 1,500 bytes. In order to observe f(p), *tcpdump* was used. Once more, the experiments were performed for the three different values of MTU: 1,500, 1,000, 500 bytes. Each experiment was held many times. Figures 8A–8C show the observed transmitted packets on one machine, for one hour.

Again, from the packets’ distribution, it is clear that most of the packets in the range of 62–88 bytes (96% of the transmitted packets). Here it is noticed that the number of 66-byte packets is increased when the MTU size is decreased. This is because more packets have to be sent and received for the same amount of data. Moreover, the acknowledgment packets will increase too (one acknowledgment packet for each received packet). In the next part, the real traffic energy consumption will be discussed.

#### Consumed energy in real traffic.

In point A, a linear equations can be derived that help in calculating the consumed energy in sending or receiving one byte for all values of MTU. These equations can be used to measure the consumed energy in real traffic. While in points B.1 and B.2, capture the packets received and sent by one machine can be captured. Therefore, by applying the previous Eqs. (3) and (5) on the captured packets, the consumed energy in real traffic can be calculated by the following generated equation:

(6) \begin{eqnarray*}E \left( m \right) =\sum _{p=1}^{m}p\ast g \left( p \right) \ast r \left( p \right) +p\ast f \left( p \right) \ast t \left( p \right) \end{eqnarray*}

Such that, the captured packets of the MTU of 1,500 bytes, the total consumed energy would be:

\begin{eqnarray*}E(1,500)=0.293~ub/hour \end{eqnarray*}

### C. MTU modification over real traffic

Since the value of MTU could not be changed for all the machines on the network, MTU transformation can be done to give an estimation of the energy consumption for different values of MTU. Graphs and packet distribution can help in doing these transformations. However, it is not useful to use the packet distribution as is. To be able to use this distribution, the distribution must be normalized to get a new distribution where the area under the curve equals 1.

The normalization is done by dividing the number of packets of all sizes, to get a normalization function *f*_*i*_(*p*), as shown in Eq. (7):

(7) \begin{eqnarray*}{f}_{i} \left( p \right) = \frac{{g}_{i} \left( p \right) }{\sum _{j=1}^{m}j\ast {g}_{i} \left( j \right) } \end{eqnarray*}

Where *i* represents the function *g* (*p*) and *f* (*p*) for a given MTU, and *j* is the size of the observed packets.

With the help of the *fi* (*p*) functions, a transformation function *τ* is achieved to estimate the global traffic for a given MTU, and the whole network. To estimate the global traffic for a given value of MTU, it is enough to apply the transformation function on the global function *g* (*p*). The idea of this algorithm is to obtain new functions *f*_*n*_ from the function *f*_1_. Noting that this transformation must preserve the quantity of traffic.

The algorithm that satisfies the previous conditions can be as the following:

\begin{eqnarray*}\tau (\mathrm{m}2,\mathrm{m}1)(\mathrm{p})= \left\{ \begin{array}{@{}l@{}} \displaystyle 0,\,\mathrm{if}~\mathrm{p}\gt \,\mathrm{m}2\\ \displaystyle f\mathit{(}p\mathit{)}+\sum _{i=m1+1}^{m1-1}f\mathit{(}i\mathit{)}\mathit{.}s\mathit{(}i,p,m2\mathit{)},\,\mathrm{if}~\mathrm{p}\gt \,\mathrm{m}2\\ \displaystyle f\mathit{(}p\mathit{)}+\mathrm{int} \left( \frac{f\mathit{(}m1\mathit{)}\mathit{\ast }m1}{m2} \right) +\sum _{i=m2+1}^{m1-1}f\mathit{(}i\mathit{)}\mathit{.}\mathrm{int} \left( \frac{i}{m2} \right) ,\mathrm{if}~\mathrm{p}=\,\mathrm{m}2\\ \displaystyle \\ \displaystyle \text{special}~\text{cases}:\\ \displaystyle \\ \displaystyle f\mathit{(}p\mathit{)}=f\mathit{(}p\mathit{)}+1,\mathrm{if}~\mathrm{p}\,=\,(\mathrm{f}(\mathrm{m}1)\mathrm{ \ast m}1)\,\text{%}\,\mathrm{m}2\\ \displaystyle f\mathit{(}66\mathit{)}=\mathrm{int} \left( \frac{f\mathit{(}66\mathit{)}\mathit{\ast }m1}{m2} \right) ,\,~\mathrm{to}~\text{represent}~\mathrm{the}~\mathrm{ack} \end{array} \right. \end{eqnarray*}

Where:

\begin{eqnarray*}\mathbi{s} \left( \mathrm{i},\mathrm{p},\mathrm{m}2 \right) = \left\{ \begin{array}{@{}l@{}} \displaystyle \mathrm{0},\hspace*{20.00003pt}\mathrm{if}~\mathrm{p}\hspace*{2.22198pt}\not = \hspace*{2.22198pt}\mathrm{i}\text{%}~\mathrm{m}\mathrm{2}\hspace*{10.00002pt}\\ \displaystyle \mathrm{1},\hspace*{20.00003pt}\mathrm{if}~\mathrm{p}=\hspace*{2.22198pt}\mathrm{i}\text{%}~\mathrm{m}\mathrm{2}\hspace*{10.00002pt} \end{array} \right. \end{eqnarray*}

*m*_1_ is the old value of MTU,

*m*_2_ is the new value of MTU

This algorithm can be summarized in the following points:

(1) The size of the new MTU must be less than the size of the old MTU. More information exists to derive a new distribution for smaller MTUs. Nevertheless, the process in the opposite way is more difficult (not done).
(2) In the new distribution, the packets that are greater than the size of the new MTU were eliminated. However, they will be divided by the new MTU size. The resulting integer quotient and the remainder are counted as new packets and added to the packets of smaller size
(3) The division process is based on multiplying the packets that have a size greater than the new MTU, by its number. The resulting value is divided by the size of the new MTU. The modal of this division is added as one packet to its equivalent packet size.
(4) Since it is known that the number of packets will increase, the number of acknowledgments must be also increased. As shown in point B.2, it is the packets of 66-byte size.

**Figure 9:**
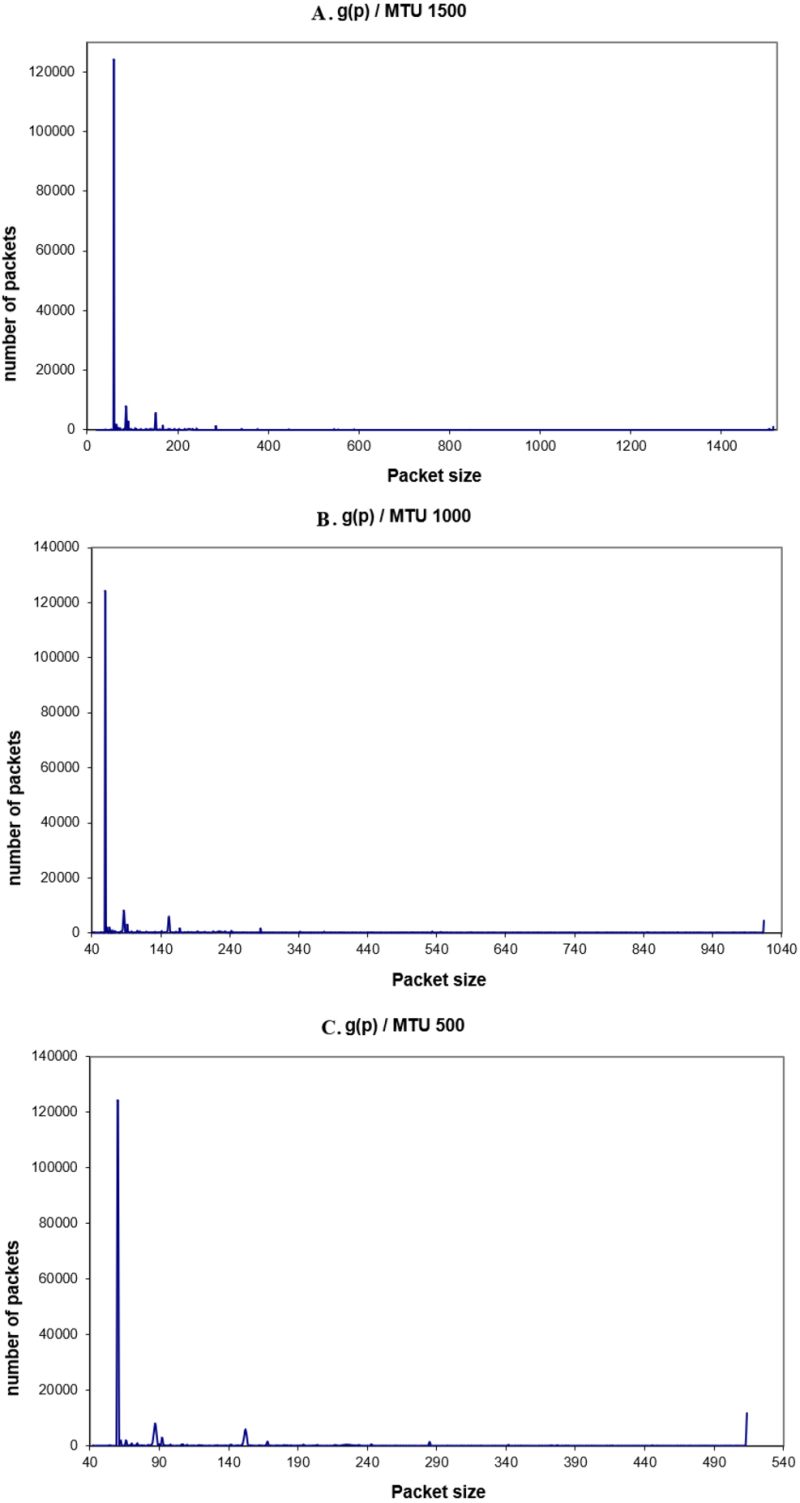
(A–C) G(p) for different values of MTU.

Figs. 9A–9C show the generated global traffic for different values of MTU.

### D. Energy consumption estimation as a function of MTU

After being able to obtain the global traffic for a given MTU, the energy consumption will be estimated now as a function of MTU based on: the generated traffic (part C), the results for the transmitted packets (part B.2), and the energy consumed (part B.3). This estimation can be calculated by using Eq. (6).

**Figure 10:**
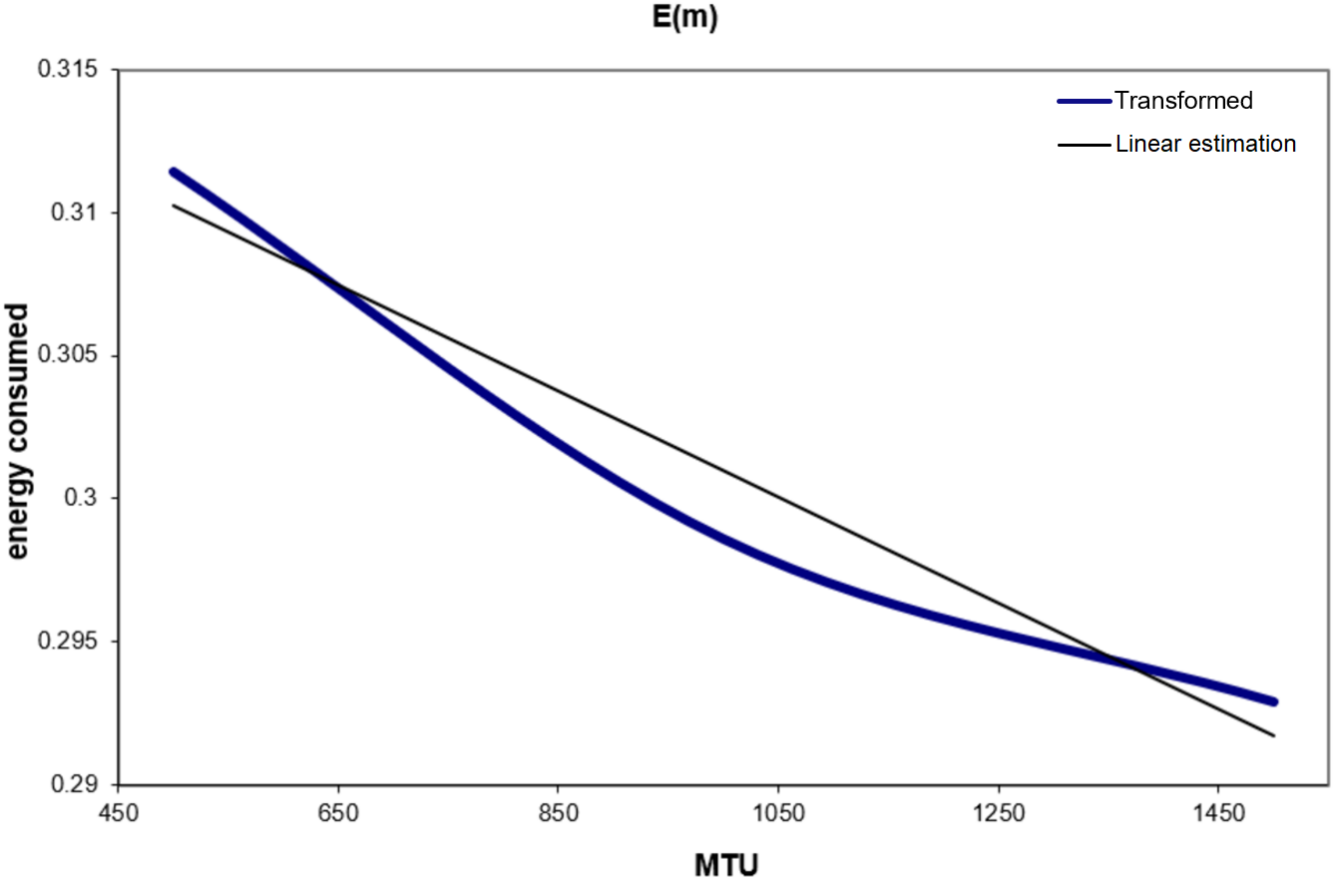
Estimation of the consumed energy.

Table 6 and Fig. 10 show the energy consumption estimation for different values of MTU after applying Eq. (6) to the results obtained. From Fig. 10, a linear equation can be obtained for the consumed energy in the function of MTU:

(8) \begin{eqnarray*}E(m)=-1.852\ast 1{0}^{-5}\ast m+0.3195.\end{eqnarray*}

Finally, with the help of Eq. (8), the energy consumed in transmission and reception by the machine can be estimated, whatever the MTU size was in the network.

## Conclusion

In this research, an original model is developed for optimal energy consumption in Fog of IoT wireless networks through optimizing the TCP protocol MTU size. Since Fog, Edge and MEC are currently widely speeded networks and they have many devices with limited energy sources, it seems evident that the energy consumption optimization in these networks plays a vital role in their durability. Our work presented the effect of the MTU size on the consumed energy. It was clear that the MTU size is inverse proportional to the amount of the consumed energy. Also, the work showed that with the help of transformation techniques it is possible to estimate the consumed energy if the network parameters were changed totally. Moreover, the work will continue to add the modification made to the existing works in the MAC layer to reduce the consumed energy in the wireless networks which in turn extend the battery life of wireless network devices.

**Table 6:** Consumed energy for different values of MTU.

**MTU**	**Consumed energy (ub/hour)**
500	0.31144272
1000	0.29861507
1500	0.29292561

##  Supplemental Information

10.7717/peerj-cs.653/supp-1 Supplemental Information 1 Data analysis

## Additional Information and Declarations

### Data Availability

The following information was supplied regarding data availability: The data are available in the Supplementary Files.
